# Gamma-radiation of *Glossina palpalis gambiensis* revisited: effect on fertility and mating competitiveness[Fn FN1]

**DOI:** 10.1051/parasite/2023009

**Published:** 2023-03-31

**Authors:** Soumaïla Pagabeleguem, Oumar Koughuindida, Ernest Wendemanegde Salou, Geoffrey Gimonneau, Ange Irénée Toé, Bénéwendé Aristide Kaboré, Kiswend-sida Mikhailou Dera, Hamidou Maïga, Adrien Marie Gaston Belem, Gisèle Marie Sophie Sanou/Ouédraogo, Marc JB Vreysen, Jeremy Bouyer

**Affiliations:** 1 Institut des Sciences de l’Environnement et du Développement Rural, Université de Dédougou (UDDG) BP 176 Dédougou Burkina Faso; 2 Insectarium de Bobo-Dioulasso – Campagne d’Éradication de la mouche Tsé-tsé et de la Trypanosomose (IBD-CETT) BP 1087 Bobo-Dioulasso Burkina Faso; 3 Insitut du Développement Rural (IDR), Université Nazi Boni (UNB) BP 1091 Bobo-Dioulasso Burkina Faso; 4 Unité de Formation et de Recherche en Sciences de la Vie et de la Terre (UFR-SVT), Université Nazi Boni (UNB) BP 1091 Bobo-Dioulasso Burkina Faso; 5 Centre International de Recherche-Développement sur l’Élevage en zone Subhumide (CIRDES) BP 454 Bobo-Dioulasso Burkina Faso; 6 Centre de Coopération Internationale en Recherche Agronomique pour le Développement (CIRAD) Montpellier 34398 France; 7 Institut Sénégalais de Recherches Agricoles, Laboratoire National d’Élevage et de Recherches Vétérinaires Service de Bio-écologie et Pathologies Parasitaires BP 2057 Dakar – Hann Sénégal; 8 Insect Pest Control Laboratory, Joint FAO, IAEA Programme of Nuclear Techniques in Food and Agriculture, International Atomic Energy Agency Wagramer-strasse 5 PO Box 100 Vienna A-1400 Austria; 9 Institut de Recherche en Sciences de la Santé BP 545 Bobo-Dioulasso Burkina Faso

**Keywords:** Tsetse flies, Sterile insect technique, Mating performance, Radiation dose

## Abstract

African animal trypanosomoses are vector-borne diseases that cause enormous livestock losses in sub-Saharan Africa, with drastic socio-economic impacts. Vector control in the context of an area-wide integrated pest management program with a sterile insect technique component requires the production of high-quality sterile male tsetse flies. In our study, we evaluated the effect of irradiation on the fecundity of *Glossina palpalis gambiensis* to identify the optimal dose that will induce maximum sterility while maintaining biological performance as much as possible. In addition, male mating performance was evaluated in semi-field cages. The irradiation doses used were 90, 100, 110, 120, 130, 140, and 150 Gy, and untreated males were used as the control. The results showed that pupal production and emergence rates were higher in batches of females that had mated with fertile males than in those that had mated with irradiated males with any experimental dose. A dose of 120 Gy administered to male flies induced 97–99% sterility after mating with virgin females. For the semi-field cage experiments, males irradiated with 120 Gy showed good sexual competitiveness as compared to fertile males and those irradiated with 140 Gy, considering the level of filling of spermatheca and the number of pairs formed. The optimal radiation dose of 120 Gy found in this study is slightly different from the traditional dose of 110 Gy that has been used in several eradication programmes in the past. The potential reasons for this difference are discussed, and an argument is made for the inclusion of reliable dosimetry systems in these types of studies.

## Abstract (fr)

Les trypanosomoses animales africaines sont des maladies à transmission vectorielle qui causent d'énormes pertes de bétail en Afrique subsaharienne, avec des impacts socio-économiques importants. La lutte antivectorielle dans le cadre d'un programme de lutte intégrée contre les ravageurs à l'échelle d’une zone avec une composante de technique d'insectes stériles nécessite la production de glossines mâles stériles de haute qualité. Dans notre étude, nous avons évalué l'effet de l'irradiation sur la fécondité de *Glossina palpalis gambiensis* afin d'identifier la dose optimale qui induira une stérilité maximale tout en maintenant au maximum les performances biologiques. De plus, les performances d'accouplement des mâles ont été évaluées en cages de semi-terrain. Les doses d'irradiation utilisées étaient de 90, 100, 110, 120, 130, 140 et 150 Gy, et des mâles non traités ont été utilisés comme contrôle. Les résultats ont montré que les taux de production et d'émergence de pupes étaient plus élevés dans les lots de femelles qui s'étaient accouplées avec des mâles fertiles que dans les lots de celles accouplées avec des mâles irradiés, avec n'importe quelle dose expérimentale. Une dose de 120 Gy administrée à des mouches mâles a induit une stérilité de 97 à 99 % après accouplement avec des femelles vierges. Pour les expériences en cages de semi-terrain, les mâles irradiés à 120 Gy ont montré une bonne compétitivité sexuelle par rapport aux mâles fertiles et à ceux irradiés à 140 Gy, en considérant le niveau de remplissage de leur spermathèque et le nombre de couples formés. La dose de rayonnement optimale de 120 Gy trouvée dans cette étude est légèrement différente de la dose traditionnelle de 110 Gy qui a été utilisée dans plusieurs programmes d'éradication dans le passé. Les raisons potentielles de cette différence sont discutées et un argument est avancé pour l'inclusion de systèmes de dosimétrie fiables dans ce type d'études.

## Introduction

Tsetse flies are hematophagous insects found in sub-Saharan Africa and are the cyclical vectors of trypanosomes, the causative agents of Human African Trypanosomosis (HAT), also known as sleeping sickness and African Animal Trypanosomosis (AAT) or nagana [8, 63]. The debilitating disease AAT constitutes a major constraint to livestock production and the development of more efficient and sustainable livestock systems in 38 sub-Saharan Africa countries [35]. In addition, it limits the exploitation of fertile land for agricultural activities in an area of 10 million km^2^ [14]. The direct annual production losses of cattle in terms of decreased meat and milk production, abortions, etc., and the cost of AAT control are estimated at USD 600–1200 million [30]. The overall annual losses in livestock and crop production have been estimated to be as high as USD 4750 million [7]. There are limited options to manage AAT through the control of the parasite itself due to the absence of efficient preventative vaccines [20] and widespread chemoresistance against the most commonly used trypanocidal drugs [16, 27]. Thus, tsetse control remains the most cost-effective option for managing AAT [24].

The management of these diseases through area-wide integrated pest management (AW-IPM) approaches of the tsetse flies using the sterile insect technique (SIT) has shown its effectiveness on Unguja Island of Zanzibar against *Glossina austeni* [61], and in the Niayes of Senegal against a *Glossina* *palpalis* *gambiensis *population [19, 43, 64]. The programs in Burkina Faso against *Glossina morsitans submorsitans*, *G*. *palpalis* *gambiensis*, and *Glossina* *tachinoides* [10, 47], and in Nigeria against *Glossina palpalis palpalis* [53] were able to eradicate the populations in the targeted areas, but suffered from re-invasion from neighbouring populations as the approach was not area-wide [38].

To date, the sustainable removal of a *G. austeni* population from Unguja Island of Zanzibar in the 1990’s remains the most successful AW-IPM program with an SIT component against a tsetse fly population. This successful program also played a catalytic role in the establishment of a Pan-African Tsetse and Trypanosomosis Eradication Campaign (PATTEC), a political initiative that aims at increasing efforts to address the tsetse and trypanosomosis problem on the African continent [37].

The SIT requires mass-rearing of the target species in a facility, followed by sterilization of the produced males using ionizing radiation, and sustained and sequential release of the sterile males over the target area in numbers large enough to outcompete the wild males for mating with wild females [5, 38, 63]. During mating, wild virgin females are inseminated with sterile sperm that after fertilization of the egg, results in embryonic death. The absence of progeny will lead to a reduction in the size of the targeted population and, in some cases, to complete local eradication [22]. The quality of the released sterile males is one of the key parameters that defines the efficiency of a program [62]. Releasing low quality sterile males will require higher release rates and might prolong the duration of the program, which itself then requires further funding, and potentially leads to program failure [58].

High doses of ionizing radiation that cause sterility in the male sperm, also damage somatic cells and this can reduce the mating competitiveness of the released males [22]. Several studies have been carried out to assess the effect of gamma radiation on the reproduction and competitiveness of different tsetse species [5, 12, 13, 33, 54, 56, 60] to determine the optimal radiation dose to induce an acceptable level or complete sterility in these species. In the eradication campaign against *G. palpalis gambiensis* in the Sidéradougou area of Burkina Faso, the optimal dose of irradiation was identified as 110 Gy using Cs^137^ [54]. This study was carried out in 1976 [54] and the radiation dose of 110 Gy has become the standard dose to be administered to adult *G*. *palpalis gambiensis* males that induces high sterility in mated females. Since then, no updated studies have been carried out to assess the impact of this dose on the competitiveness of the males. There have been other studies where *G. palpalis gambiensis* adults or pupae were irradiated with 110 Gy, but these studies were carried out to assess the effects of transport and release conditions on the quality of sterile males [18, 41, 43, 50].

The “Insectarium of Bobo-Dioulasso” (IBD) has the capacity to produce about 1,000,000 sterile male tsetse per week, and aims at supporting various control campaigns in the West African sub-region [44, 46]. Rearing of *G. palpalis gambiensis* started in June 2016 at the IBD, and since 2017 the facility has supplied around 8 million sterile male pupae for the AW-IPM program in the Niayes of Senegal [44]. The pupae were irradiated with 120 Gy using a Co^60^ FOSS 812 irradiator. It was deemed necessary to investigate the optimal irradiation dose for *G*. *palpalis gambiensis*, particularly as local conditions can impact the efficiency of irradiation, including dose-rate [65]. This study was therefore initiated to assess the effect of gamma-radiation on *G. palpalis gambiensis* male pupae, as well as their mating performance in walk-in field cages.

## Materials and methods

### Insectary

The study was carried out at the “Insectarium de Bobo-Dioulasso” (IBD), located in Darsalamy, 15 km from Bobo-Dioulasso (11°03′32.4″ N and 4°21′10.9″ W), Burkina Faso. The IBD is a facility that specializes in mass-rearing of tsetse flies. It was built under the auspices of the PATTEC initiative to satisfy the needs in sterile male tsetse flies for the PATTEC program in Burkina Faso and other countries infested by tsetse flies. There are currently two species mass-reared at the IBD: *G. palpalis gambiensis* and *G. morsitans submorsitans*. The rearing rooms are completely climate controlled in terms of air circulation, cooling and humidification, i.e., a temperature of 25 ± 1 °C, relative humidity of 75 ± 5% RH and a 12:12 h light:dark photoperiod [26]. The IBD houses a Co^60^ irradiator (FOSS model 812 SN 002) that has two sources of initially 7500 Curie, and that is used to sterilize male flies and blood.

### Experimental pupae

All pupae of *G. palpalis gambiensis* used for this study were obtained from the laboratory colony maintained at the IBD. The colony was derived from a strain that was established at Maisons-Alfort, France in 1972, which originated from Guinguette, a locality near Bobo-Dioulasso, Burkina Faso. It was transferred to the Centre de Recherche sur les Trypanosomiases Animales (CRTA), Burkina Faso in 1975 [4, 34, 51] [CRTA is the former name of the Centre International de Recherche-Développement sur l’Élevage en zone Subhumide (CIRDES)]. In 2016, 53,972 adult flies from this CIRDES colony were transferred to the IBD where a colony was likewise established for mass-rearing. The flies were fed on abattoir-collected defibrinated bovine blood using an artificial *in vitro* membrane feeding system.

### Irradiation of pupae

Batches of pupae from which almost all females have emerged (about three days after the beginning of emergence) were chilled at 8 °C and then irradiated at the same temperature with the experimental dose. In total, 2400 male pupae were sampled and divided into eight batches. Seven batches of pupae were irradiated with doses of 90, 100, 110, 120, 130, 140 and 150 Gy using one Co^60^ source of the FOSS 812 irradiator at a dose rate of 37.74 Gy/min. The eighth batch was not irradiated and served as a control. After irradiation, the male pupae were put in a petri dish that was placed in a PVC tube (10 cm high and 8.4 cm in diameter), and this set-up was placed in an emergence cage (30 × 25 × 15 cm). The purpose of this setup was to select only flying males for the experiments, i.e., males that managed to escape the PVC tube after emergence, ending up in the emergence cage [50].

### Effect of irradiation on reproduction

Seven-day-old irradiated and untreated virgin males were mated with four-day-old virgin untreated females at a male to female ratio of 1:1 and put in Roubaud cages (13.5 × 8 × 4.5 cm). For each treatment dose, five Roubaud cages containing 30 males and 30 females were used, and each cage corresponded to a single repetition. Mating cages were placed in individual larviposition cups and pupae were collected daily (except Sundays) and sorted into viable and aborted larvae. The viable pupae were weighed using an electronic balance with a sensitivity of 0.0001 mg and with automatic calibration (Sartorius MSE2 7S-000-DM Cubis Ultra) and classified according to their weight (mg): *A* < 22, 22 ≤ *B* < 28, 28 ≤ *C* < 32, 32 ≤ *D* < 36, *E* ≥ 36 [25]. The pupae were incubated at 25 ± 1 °C, and 75 ± 5% RH until adult emergence.

For each treatment, the rate of induced sterility was calculated as follows: the number of pupae produced per reproductive female and per day (from day 18 post-emergence) [54]. At the end of the experiments (60 days), 30 surviving females from each treatment dose were dissected to determine their reproductive status, insemination rate and spermathecal fill [67]. In each case, the content of the uterus was examined to record the presence/absence of eggs or larvae. The spermathecae were extracted and their filling rate was evaluated under a microscope.

### Male mating performance

Following the evaluation of the effects of irradiation on the productivity of *G. palpalis gambiensis*, the doses of 120 and 140 Gy were selected for the study on male mating performance. The study was conducted in walk-in field cages [39] that were deployed outside next to the insectarium. The walk-in field cage consisted of a cubic wooden frame (2.9 × 2.9 × 2 m) covered with a mosquito net, and with a *Citrus limon* plant, about 1.5 m high, placed centrally in the cage. Access to the cage was controlled by a double zipper that could be closed from the inside and outside. Throughout the experiment, temperature and relative humidity were recorded every 10 min using a Hobo U14-001 data logger, and the light intensity at the top and bottom of the cage and at tree level was recorded using a Testo 540 light meter.

Three groups of males were used: a group irradiated with 120 Gy, a second irradiated with 140 Gy, and a third that was not irradiated. Upon emergence, male pupae were placed in petri dishes and covered with ~1 cm of sterile sand mixed with a fluorescent dye (DayGlo) (0.5 g dye/200 g of sand) to mark the males during emergence to allow differentiation between the three male groups [50]. The test was replicated nine times.

The field cage experiments were carried out in the morning from 07:30 to 10:30 local time, when environmental conditions were favorable. Thirty virgin females, aged three days, were released at the center of the cage, and 10 min later 90 six-day-old males were released, i.e., 30 males from each group, giving a female to male ratio of 1 to 3. The non-fliers (males and females) were replaced a few minutes after the beginning of the experiment to obtain 30 females and 30 operational males for each group.

Throughout the three-hour period of each experiment, an observer remained inside the cage and movements were kept to a minimum. Each mating pair was collected and put into an individual vial. The time of the initiation of the mating was recorded for each pair to determine mating latency, and the duration of the mating was also recorded for each pair. At the end of the experiment, the mated females were dissected to assess insemination rate and spermathecae fill. For each mated female, information on mating latency time, mating duration, insemination rate and spermathecae fill was available. The males of each mating pair were examined under an ultraviolet camera [50] to identify their color mark, and to determine the rank and number of males mated from each group. All flies remaining in the cage at the end of the observation period were recaptured, with unmated females dissected to confirm their virginity and the remaining males discarded.

The mating performances of the males in the three groups were compared using the following indices: mating propensity (MP), defined as the overall proportion of released females that had mated, relative mating index (RMI), defined as the number of pairs of one treatment group as a proportion of the total number of mating pairs, and relative mating performance (RMP), defined as the difference between the number of mating pairs of two treatments of males as a proportion of the total number of mating pairs [39].

### Statistical analysis

R Software (version 4.0.3) [48] with RStudio [49] (Boston, MA, USA) was used for the statistical analyses. The Shapiro-Wilk test was used to test the normality of data and the Tukey’s test was applied.

For female fertility, the generalized linear mixed effect model was used to analyze the number of pupae produced per female every 10 days (pf10d) (with a Poisson distribution), the mean pupal weight (with a Gaussian distribution), and emergence rates (with a binomial distribution) [3], with the dose considered as a fixed variable. Differences between the levels of significant fixed factors were analyzed using post hoc Tukey tests (glht function in package *multcomp*) [6].

The relative mating index, average mating latency, mating duration, insemination rate, and spermathecal fill were compared between treatments (0, 120 and 140 Gy) using the generalized linear mixed effect model with the dose considered as a fixed variable. The data analysis details are available as a supplementary file (S1 Data).

## Results

### Productivity of females

**Figure 1:**
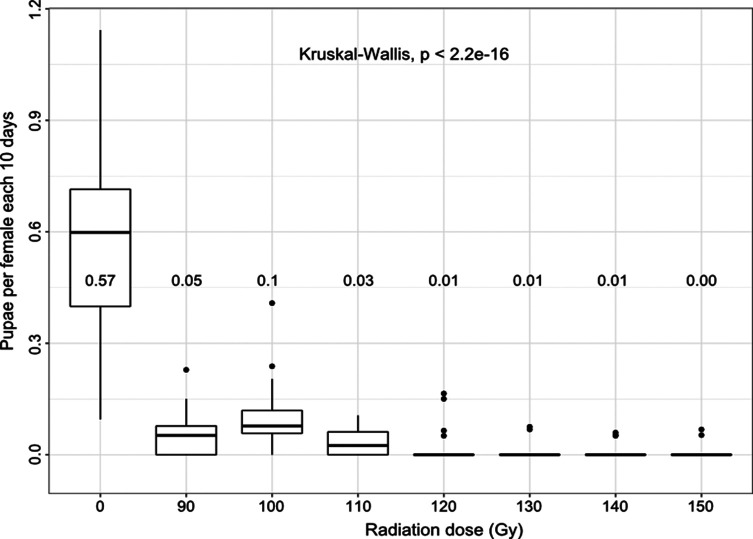
Average number of pupae produced every 10 days per female mated with males irradiated with different doses. Values in the figure correspond to the means.

On day 18 post-emergence (approx. the first larviposition date), survival of the females was similar between treatments (Table 1). Fecundity (number of pupae produced per female every 10 days) was dose-dependent and decreased significantly with increasing radiation doses (*p* < 0.001; Fig. 1). A negative linear regression was observed between the fecundity of females that had mated with irradiated males and the dose of irradiation (*r* = −0.84; *p* < 0.001), i.e., females mated with males irradiated with doses ranging from 90 to 150 Gy (with an increment of 10) produced 29 to 2 pupae, representing a production level of 11.6% to 0.8% as compared with females mated with untreated males (*n* = 251 pupae). The induced sterility was 89, 80, and 93% for females mated with males irradiated with 90, 100, and 110 Gy, respectively. A dose of 120 to 150 Gy administered to males induced 97–99% sterility in untreated females mated with them (Table 1).

**Table 1:** Pupal production parameters of *G. palpalis gambiensis* females mated with males irradiated with different doses after 60 days monitoring.

Dose (Gy)	Replications	No. of mature females (18 days)	No. pupae produced	Classification of pupae according to their weight (mg)					%Emergence/%female	Induced sterility (%)
				A (%)	B (%)	C (%)	D (%)	E (%)		
				<22	22–<28	28–<32	32–<36	>36		
0	5	133	267^a^	39 (14.61)	178 (66.67)	46 (17.23)	4 (1.50)	0	98.13/55.73	0
90	5	135	27^b^	3 (11.11)	19 (70.37)	5 (18.52)	0	0	74.07/50	89
100	5	138	49^b^	7 (14.29)	29 (59.18)	11 (22.45)	2 (4.02)	0	81.63/47.5	80
110	5	130	16^b^	0	11 (68.75)	5 (31.25)	0	0	75/50	93
120	5	125	7^b^	3 (42.86)	4 (57.14)	0	0	0	71.43/20	97
130	5	133	2^b^	1 (50)	1 (50)	0	0	0	50/50	99
140	5	134	4^b^	2 (50)	2 (50)	0	0	0	50/50	98
150	5	127	2^b^	1 (50)	1 (50)	0	0	0	0/0	99

In a column, the values that have a common letter (a or b) are not significantly different (*p* > 0.05).

The number of pupae produced during the 60-day trial was higher in females that had mated with fertile males than in those that had mated with irradiated males with any experimental dose (Table 1). Most of the pupae produced by females that had mated with males irradiated with a dose lower than 110 Gy were in pupal mass class B or above, similar to the class of pupae produced by females that had mated with fertile males (*p* > 0.1; Table 1). However, females that had mated with males irradiated with a dose greater than 110 Gy produced pupae belonging to pupal mass class B or lower, smaller than the pupae produced by females that mated with fertile males (*p* < 0.01; Table 1).

Adult emergence from pupae produced by females that had mated with fertile males (98.13%) was greater than emergence of pupal batches of females that had mated with irradiated males irrespective of the dose (less than 82%) (*p* < 0.01; Table 1). There was no difference in emergence of pupal batches from females that had mated with sterile males irradiated at different doses. The number of pupae produced (and subsequent adult emergence) by females that had mated with males irradiated with the high doses (130, 140, and 150 Gy) was insufficient for multiple comparisons (i.e., 2, 4, and 2 pupae incubated for 130, 140, and 150 Gy, respectively corresponding to 1, 2, and 0 emerged adults). 

Dissection of females at the end of the experiments on day 60 post emergence, showed an insemination rate of 100% in all batches, except the females that had mated with males irradiated with 150 Gy (insemination rate of 93.3%) (Table 2). Spermatheca fill was influenced by radiation dose (*p* < 0.001) and varied between 85 and 93% for females in the control batch, as well as for the females that had mated with males that had been irradiated with a dose ≤ 130 Gy. Spermathecal fill was 75 and 73% for the 140 and 150 Gy treatment group, respectively (Table 2).

**Table 2:** Dissection results of *G. palpalis gambiensis* females mated with males irradiated with different doses.

Doses	No. of females dissected	Insemination%	Spermathecal fill	Abortion		Contents of the uterus				
					ovulated egg	Empty (%)	Degenerated egg	Instar larval		
								L1	L2	L3
0	30	100	86.25 ± 23.75	0	30	2 (6.67)	0	10	8	10
90	30	100	92.92 ± 18.18	2	28	8 (26.67)	21	0	0	1
100	30	100	84.17 ± 17.96	1	29	6 (20)	24	0	0	0
110	30	100	90.83 ± 17.66	1	29	7 (23.33)	23	0	0	0
120	30	100	84.58 ± 26.20	4	26	12 (40)	18	0	0	0
130	30	100	89.58 ± 21.30	3	27	8 (26.67)	22	0	0	0
140	30	100	75.00 ± 26.26	7	23	12 (40)	18	0	0	0
150	30	93.33	72.92 ± 29.56	3	27	6 (20)	24	0	0	0

L1: 1st larval stage, L2: 2nd stage larval, L3: 3rd larval stage.

**Figure 2:**
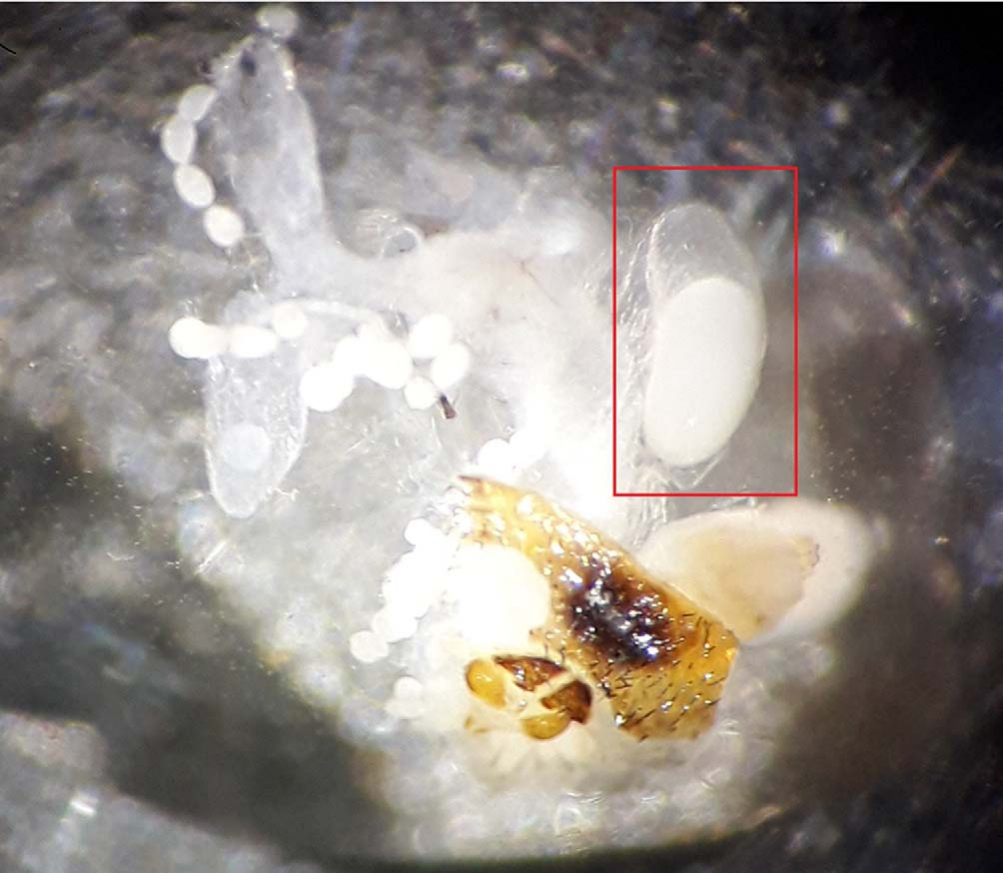
Degenerated egg (framed in red).

The reproductive status of females that had mated with the untreated males was markedly different from that of females that had mated with irradiated males regardless of dose (Table 2). Females that had mated with fertile males had an empty uterus due to recent larviposition, or a uterus that contained a recently ovulated egg or a viable larva. Females that had mated with irradiated males had either an empty uterus due to abortion, or a uterus that contained a newly ovulated egg or a degenerating egg (in embryonic arrest) (Fig. 2), except for one female that had mated with a male irradiated at 90 Gy, whose uterus contained one L3 larva (Table 2).

**Figure 3:**
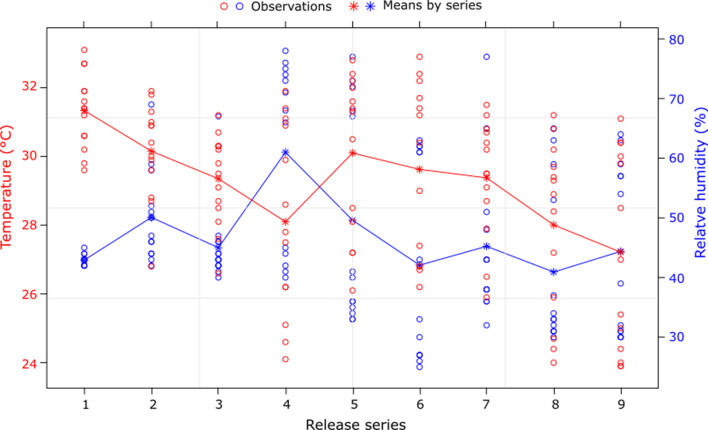
Variation in temperature and relative humidity in the field cage during the assessment of the mating performance of irradiated males.

### Male mating performance

#### Environmental conditions in field cage

The field cage experiments to assess mating performance of males irradiated with different irradiation doses were carried out during the first two weeks of November 2020. The mean temperature recorded in the cage was 29.27 ± 2.40 °C and the mean relative humidity was 46.76 ± 13.75%. During the study period, the relative humidity remained stable, except on the fourth day when it was higher, whereas the temperature showed a downward trend over time (Fig. 3). The light intensity ranged from an average of 1481.65 ± 955.83 Lx at the top of the experimental cage to 671.39 ± 198.29 Lx at the bottom.

#### Male mating performance

A total of 1,080 flies were released during the study: 270 females and 810 males (270 non-irradiated, 270 irradiated with 120 Gy, and 270 irradiated with 140 Gy) and 124 mating pairs were collected out of 270 potential couples, i.e., a total mating propensity of 45.92%.

The untreated males formed more mating pairs than irradiated males; however, the average relative mating index (RMI) for the untreated males (0.52) was similar to that for males irradiated with 120 Gy (RMI = 0.33; *p* = 0.23), but significantly different to that for males irradiated with 140 Gy (RMI = 0.15; *p* < 0.001, Table 3). The average relative mating index between males irradiated with 120 and 140 Gy was also significantly different (*p* = 0.02; Table 3).

**Table 3:** Assessment of the competitiveness of male *G. palpalis gambiensis* in the field cage.

Dose	Relative mating index	Mating latency time (min)	Mating duration (min)	Insemination (%)	Spermathecal fill (%)
0	0.52^a^	50.64 ± 52.96^a^	53.20 ± 22.88^a^	100^a^	85.28 ± 22.69^a^
120	0.33^a^	49.93 ± 44.79^a^	50.15 ± 25.13^ab^	97.56^a^	71.19 ± 28.54^b^
140	0.15^b^	65.16 ± 53.76^a^	39.05 ± 18.49^b^	94.74^a^	75.79 ± 28.41^ab^

In a column, the values that have a common letter (a or b) are not significantly different (*p* > 0.05).

The latency time (time between release of males and formation of mating pairs) did not differ statistically between batches of males (df = 2, *p* = 0.3; Table 3), and ranged from 1 min after the release of males to 170 min. More than half of the mating pairs were formed during the first hour after the release of the males (65.32%), 20.97% during the second hour, and 17.71% during the third hour. Mating pairs formation decreased with increasing temperatures in the field cage during the assessment, but the correlation was not significant (*p* = 0.34; cor = −0.16). Males irradiated with a dose of 120 Gy (49.93 ± 44.79 min) mated on average sooner than untreated males (50.64 ± 52.96 min) and males irradiated with a dose of 140 Gy (65.16 ± 53.76 min). Males that had a short latency time mated on average longer and vice versa (Table 3). Mating times were different between treatments (*p* = 0.04). A multiple comparison indicated that mating time was longer for untreated males than those irradiated with 140 Gy (*p* = 0.03) and similar among other groups (0 and 120 Gy, *p* = 0.7; 120 and 140 Gy, *p* = 0.2).

Examination of the reproductive systems of mated females showed that the insemination rate was above 95% with a spermathecae fill above 71%. Both parameters were higher with females that had mated with untreated males than those that had mated with irradiated males (Table 3).

## Discussion

In support of the PATTEC initiative, two tsetse mass-rearing facilities were constructed in sub-Saharan Africa, i.e., in Burkina Faso and in Ethiopia. Each of these facilities has a capacity to produce one million sterile male tsetse flies per week, i.e., *G. palpalis gambiensis* in Burkina Faso and *Glossina pallidipes* and *Glossina fuscipes fuscipes* in Ethiopia. The goal of these mass-rearing facilities is to support the implementation of the SIT component of AW-IPM programs in Africa. Since 2017, the IBD has supplied around 8 million sterile male *G. palpalis gambiensis* pupae in support of the AW-IPM program in the Niayes of Senegal [44].

The first eradication campaign against *G. palpalis gambiensis* was implemented in the 1980’s in the Sidéradougou area of Burkina Faso [47]. The male flies for this program were sterilized using a Cs^137^ irradiator and a dose of 110 Gy was used based on a study by Tazé et al. [54]. This dose has been used ever since as a reference for irradiation levels, irrespective of whether pupae or adults were irradiated. This is important given that tsetse pupae are more radiosensitive than adults [13, 56]. It was therefore deemed necessary to re-assess the validity of the 110 Gy dose, especially as local conditions can impact the efficiency of irradiation, including dose [65].

Not only are dose-response studies required to select an appropriate dose but the mating competitiveness of the male insects produced must be assessed [45, 58]. A dose below the optimal will result in insects that are not sufficiently sterile, and too high a dose may result in the release of insects that are not sufficiently competitive with wild flies [45].

### Pupal production

Between 80 to 99% sterility was induced in females that had mated with males irradiated with a dose of 90 to 150 Gy. Sterility showed a dose-dependent increase with dose. Our results are in agreement with previous studies with *G. palpalis gambiensis* [54] and with other tsetse species [13, 55, 56, 60], i.e., the amount of dominant lethal mutations introduced in the sperm of tsetse flies when exposed to radiation as expressed by induced sterility in the female mates, increased with increasing dose. In the present study, the lowest radiation dose that induced sterility rates of 97 to 99% was obtained with 120 Gy, and was considered optimal as it exceeded the threshold of 95% [57]. This irradiation dose is higher than the 110 Gy previously proposed [54], a dose that was used in the program that eradicated *G. palpalis gambiensis* from the Sidéradougou pastoral zone in Burkina Faso [10, 47], which was later reinfested from neighboring areas [38]. Previous studies on the irradiation of other *Glossina* species with a 120 Gy dose showed about 95% sterility for *G. tachinoides* [60], approximately 97 and 98% sterility for *G. palpalis palpalis* [55] and total sterilization for *G. austeni* [11, 61].

Studies with *G. austeni *[13], and *G. palpalis palpalis* [56] showed that pupae are more radiosensitive than adults. Similar results were obtained with *Glossina brevipalpis*, i.e., irradiating pupae with 40 Gy induced 97% sterility versus 93% sterility in case the dose was administered to adults [12]. This is due to the extensive development and cell division during the pupal stage as compared with the adult stage, because cells are more susceptible to radiation damage during periods of rapid division [36]. The historical data of irradiating adult *G. palpalis gambiensis* and the data of our study seem slightly different, i.e., a dose of 110 Gy administered to adult males induced 95% sterility in females that had mated with them [54], whereas in our study, 93% sterility was induced in the females with irradiation carried out at the pupal stage with the same dose. Although both studies were carried out with the same strain of *G. palpalis gambiensis*, the radiation source (Cesium 137 GAAA^®^ irradiator versus Cobalt 60 FOSS irradiator), the dose rate (10 Gy/min versus 37.74 Gy/min), and the period of strain domestication (5 years versus 50 years) were different. Fortunately, we did not observe any impact on the eradication program in Senegal, which might be related to the fact that low residual fertility is not problematic when applying SIT, provided that it is not too high (which is the case at 110 Gy) and that it is compensated by increased competitiveness of the sterile males.

It is possible that the observed difference in induced sterility might have been related to these factors. Although some earlier studies with *Aedes aegypti* [23], *Bactrocera tryoni* [9] and *Dacus cucumis* [30] showed that sterility was independent of dose rate, other research showed a positive correlation between dose rate and induced sterility [28]. A recent, more detailed study with *Ae. aegypti* pupae showed that at high doses, lower dose rates achieved greater sterility than higher dose rates [65]. The difference might also be related to a different radiation protocol used. The target dose in this study was calculated using a dose-rate decreasing Excel sheet, initially developed in 2017 with an absorbed dose being within a 95% confidence interval. Therefore, the dose of 120 Gy used in this study is in essence a nominal dose that could be different from the real absorbed dose, as a reliable dosimetry system was not available to confirm this. Indeed, a reliable dosimetry system is very important to ensure that the absorbed dose is similar to the target dose [17]. A recent study showed that in radiation studies that are implemented without a dosimetry system, the calculated dose could be off target from the real absorbed dose [32]. In addition, Yamada et al. (2023) showed that a dose of 110 Gy administered to pupae by the Raycell MK2 blood irradiator, induced more than 97% sterility [66], supporting the previous result of Tazé et al. [54]. It is known that a given dose of X-rays and gamma rays yields in many instances a similar biological response [29]. More research will be required to assess more in detail the radiosensitivity of pupae versus adult *G. palpalis gambiensis. *In addition, attention should be given to other parameters such as adult emergence rate, flight propensity, and survival that are important when selecting an appropriate radiation dose. This is important as some programs will decide on adult irradiation and others on pupal irradiation. The Sidéradougou pastoral zone program used adult irradiation, in view of the proximity of the target area to the mass-rearing facility (Sidéradougou and Bobo-Dioulasso are less than 100 km apart). Pupal irradiation, however, is being used for the Senegal program, as the insects need to be shipped over 2000 km from Burkina Faso to Senegal, and this is only possible as irradiated pupae [43].

The dissection data revealed that the uterus of females that had mated with males irradiated with 120 Gy were either empty due to the abortion of the egg in embryonic arrest, or contained a recently ovulated egg, or an egg in embryonic arrest due to the consequences of irradiation. Our results are in line with other studies with *G. palpalis palpalis* [52] and *G. austeni* [13]. Indeed, the discrepancy between uterus content and the size of the follicle next in ovulation sequence observed in females that had mated with sterile males is a useful method to assess whether a female has mated with a sterile male or a fertile male, and hence, can be used to assess the level of induced sterility in the wild female population [59]. The method was used successfully with a high level of accuracy to assess the rate of induced sterility in the wild female *G. austeni *population during the eradication campaign on Unguja Island [61] and against *G. palpalis gambiensis* during the ongoing eradication phase in Senegal [2, 64].

### Mating performance

In this experiment, an average propensity to mate of 46% was observed, which was lower than that obtained for the same species (57%) in the same area [32]. It was also lower than the mating propensity obtained with other tsetse species such as *G.* *brevipalpis* (57%) [12], *G. austeni* (63%) [13], *G. fuscipes fuscipes* (59%), and *G. palpalis palpalis* (69%) [1] using a similar experimental set-up. The lower mating propensity in our study might be related to less optimal environmental conditions, i.e., 29.3 ± 2.4°C and 46.8 ± 13.7% as compared with the previous studies (24.4 ± 0.4 °C and 53.7 ± 1.8% [1], 25–29 °C and 50 – 94% [32], 26.1 ± 4.1°C and 47.9% ± 17.6% [13]). Optimal environmental conditions appear necessary for mating activity to take place, which may include temperature and light intensity within a certain range and adequate humidity [42]. The mating performance tests carried out using a field cage either set up outdoors in Burkina Faso or inside the laboratory in Austria showed that high temperatures in the field cage may suppress flight activity [40].

There was no significant difference between the number of pairs formed by the non-irradiated males and those irradiated with 120 Gy. Our results are in line with those observed with *G. pallidipes* in a field cage study [39]. In addition, as with De Beer et al. [12], our study showed that the males that were the fastest to form pairs were those whose mating lasted the longest. Although the number of mating pairs formed was lower with males irradiated with 120 Gy, the short latency time of these flies seems to confirm that these males are able to compete with their wild counterparts. A delay in the initial mating of an irradiated male could potentially reduce its competitiveness in the field.

The spermathecal fill of females that had mated with non-irradiated males was on average better than the spermathecal fill of females that had mated with irradiated males. Their insemination ability gradually decreased with increasing irradiation dose [54]. These results seem interesting as the doses applied did not compromise the ability to inseminate, especially for the dose of 120 Gy. Indeed, if 100% filled, a female can even not use half of her spermathecal content throughout her life [21]. The relative mating index, the mating latency time, and the mating duration were similar between non-irradiated males and males irradiated with 120 Gy. Overall, the field cage experiments and the dissection of females showed that a 120 Gy dose had no impact on the sexual vigor of the males or the insemination of the females. This is in accordance with field cage evaluations of *G. morsitans*, *G. pallidipes*, and *G. austeni* that also showed that the competitiveness of irradiated males did not differ from that of untreated males [13, 15, 39].

## Conclusion

The objective of the present study was to review the optimal dose of irradiation of *G. palpalis gambiensis* set 50 years ago. It appears that, in our conditions, male pupae irradiated with a dose of 120 Gy induced at least 97% sterility to females. The results of this study confirm the good sterility and quality of pupae sent to the Senegal eradication program, as evidenced by the satisfactory results in the field. In fact, the target tsetse population has been eliminated since 2011, with < 0.001 fly per trap per day until the end of 2018 [64]. Releases are still ongoing and elimination from the whole area is in sight.

## Supplementary materials

The Supplementary materials of this article are available at https://www.parasite-journal.org/10.1051/parasite/2023009/olm. *S1 Data*: Data analysis details.
